# An In Vitro Model for Assessing Corneal Keratocyte Spreading and Migration on Aligned Fibrillar Collagen

**DOI:** 10.3390/jfb9040054

**Published:** 2018-09-21

**Authors:** Pouriska B. Kivanany, Kyle C. Grose, Nihan Yonet-Tanyeri, Sujal Manohar, Yukta Sunkara, Kevin H. Lam, David W. Schmidtke, Victor D. Varner, W. Matthew Petroll

**Affiliations:** 1 Department of Ophthalmology, University of Texas Southwestern Medical Center, Dallas, TX 75390, USA; pouriskakivanany@gmail.com (P.B.K.); kyle.grose94@gmail.com (K.C.G.); yonetnihan@gmail.com (N.Y.-T.); sujal_manohar@hotmail.com (S.M.); yukta.sunkara@gmail.com (Y.S.); 2 Department of Bioengineering, University of Texas at Dallas, Richardson, TX 75080, USA; kevin.lam2@utdallas.edu (K.H.L.); david.schmidtke@utdallas.edu (D.W.S.); vdv@utdallas.edu (V.D.V.); 3 Department of Surgery, University of Texas Southwestern Medical Center, Dallas, TX 75390, USA

**Keywords:** corneal stroma, corneal keratocytes, wound healing, collagen fibrils, growth factors, extracellular matrix, topography, microfluidics, engineered substrates

## Abstract

Background: Corneal stromal cells (keratocytes) are responsible for developing and maintaining normal corneal structure and transparency, and for repairing the tissue after injury. Corneal keratocytes reside between highly aligned collagen lamellae in vivo. In addition to growth factors and other soluble biochemical factors, feedback from the extracellular matrix (ECM) itself has been shown to modulate corneal keratocyte behavior. Methods: In this study, we fabricate aligned collagen substrates using a microfluidics approach and assess their impact on corneal keratocyte morphology, cytoskeletal organization, and patterning after stimulation with platelet derived growth factor (PDGF) or transforming growth factor beta 1 (TGFβ). We also use time-lapse imaging to visualize the dynamic interactions between cells and fibrillar collagen during wound repopulation following an in vitro freeze injury. Results: Significant co-alignment between keratocytes and aligned collagen fibrils was detected, and the degree of cell/ECM co-alignment further increased in the presence of PDGF or TGFβ. Freeze injury produced an area of cell death without disrupting the collagen. High magnification, time-lapse differential interference contrast (DIC) imaging allowed cell movement and subcellular interactions with the underlying collagen fibrils to be directly visualized. Conclusions: With continued development, this experimental model could be an important tool for accessing how the integration of multiple biophysical and biochemical signals regulate corneal keratocyte differentiation.

## 1. Introduction

The corneal stroma is a highly ordered structure consisting of approximately 200 collagen lamellae [1]. Corneal stromal cells (keratocytes) reside between these lamellae and are responsible for secreting the extracellular matrix (ECM) components required to develop and maintain normal corneal structure and function [2,3,4]. Biophysical interactions between these cells and their surrounding ECM play a central role in corneal morphogenesis and wound healing. Cellular forces organize the ECM into tissue-specific patterns during embryonic development [5,6,7], and feedback between cell and matrix mechanics has been shown to regulate keratocyte behavior [8]. Following injury or refractive surgery in the adult cornea, wound contraction and tissue remodeling also depend on mechanical interactions between corneal keratocytes and ECM fibrils [9,10,11]. Understanding these behaviors is also important to developing new advances in the field of tissue engineering, where it is necessary to modulate cell and ECM patterning to direct the formation of specific tissue geometries [12], or to prevent cell-induced matrix disruption of pre-fabricated 3-D constructs [13].

In addition to growth factors and other soluble biochemical factors, feedback from the ECM itself can play a key role in modulating cell behavior [10,14,15,16]. While much is known regarding the effects of growth factors on keratocyte differentiation on planar rigid 2-D substrates, corneal keratocytes reside in a much more complex ECM environment in vivo, which includes a combination of mechanical and topographical cues. Small nanoscale changes in topographical parameters (i.e., height, depth, width, and spacing) can produce significant changes in cell morphology, differentiation, and migration mechanisms in a variety of cell types [17,18,19]. Aligned surface grooves have been reported to inhibit the transformation of corneal fibroblasts to myofibroblasts following culture in TGFβ [20]. They also increase the alignment of cells and matrix within self-assembled sheets derived from corneal fibroblasts stimulated with ascorbic acid [21,22,23,24,25,26]. Furthermore, tissue equivalents produced by corneal fibroblasts plated on aligned substrates are thicker, denser, and more resistant to proteolytic degradation than those plated on unaligned substrates [13]. Recent studies have identified co-alignment between keratocytes and the collagen lamellae during stromal repopulation following transcorneal freeze injury or keratectomy in a rabbit model, suggesting topographic guidance of intrastromal cell migration in vivo [14,27,28].

Together, these studies demonstrate that substrate topography can modulate the patterning and differentiation of corneal keratocytes both in vitro and in vivo. The alignment of cells and the forces they generate has been shown to impact collagen deposition and organization [21,22,29,30,31,32]. Thus, the ability to pattern cells using substrates with well-defined topography may also be critical to controlling cell and ECM patterning during tissue engineering. Previous studies describe fabrication of self-assembled aligned fibrillar collagen substrates using microfluidics [33,34]. Unlike other techniques, fibril density and collagen concentration can be easily modulated using microfluidics, and the size of the channel can be customized to the desired dimensions of the aligned collagen substrate. In addition, since microfluidics allows the collagen fibrils to be deposited on glass substrates, they can be visualized using high resolution imaging. Finally, additional protein patterns can potentially be fabricated on a single substrate.

In this study, we evaluate for the first time the effects of aligned fibrillar collagen substrates created using a microfluidics approach on corneal keratocyte morphology, cytoskeletal organization, and alignment after stimulation with PDGF or TGFβ. We also use time-lapse imaging to assess the dynamic interactions between cells and fibrillar collagen during wound repopulation following a novel in vitro freeze injury model. We demonstrate that corneal keratocytes cultured in basal serum free media tend to spread parallel to aligned collagen fibrils. Interestingly, the degree of cell/ECM co-alignment further increased when keratocytes were exposed to PDGF or TGFβ. The freeze injury model produced of an area of cell death without disrupting the organization of the collagen fibrils, thus allowing assessment of cell patterning during wound repopulation. High magnification time-lapse differential interference contrast (DIC) imaging allowed cell movement and subcellular interactions with the underlying collagen fibrils to be directly assessed. With continued development, such as the addition of other protein patterns or variations in substrate elasticity, this experimental model could be an important tool for accessing how the interplay between biophysical and biochemical signals regulate corneal keratocyte differentiation and mechanical behavior.

## 2. Results and Discussion

### 2.1. Experimental Models

**Figure 1:**
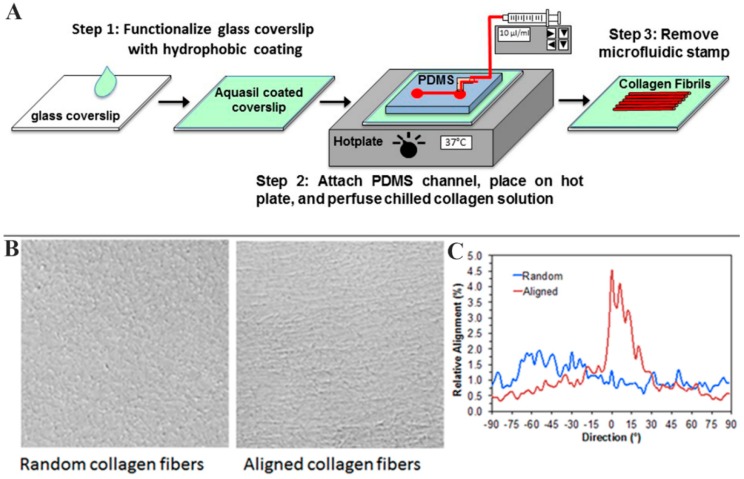
Methods and outcomes for fabrication of collagen. (**A**) Schematic of the microfluidics approach employed for producing aligned fibrillar collagen; (**B**) Differential interference contrast (DIC) images of random and aligned collagen substrates. Direction of flow during aligned collagen deposition was horizontal (0°); (**C**) Quantitative analysis of alignment of random and aligned collagen fibrils. Random fibrils showed no preferential alignment, whereas a peak in alignment near 0° was observed for the aligned collagen substrate.

Glass substrates were coated with either random (unaligned) collagen fibrils, aligned collagen fibrils created using a microfluidics device (Figure 1A), or non-fibrillar collagen. Aligned fibrils were clearly visualized using DIC microscopy (Figure 1B, right). For random substrates, collagen fibrils appeared tangled and orientated without preference to a specific direction (Figure 1B, left). Non-fibrillar collagen-coated substrates contained soluble, monomeric collagen, where no collagen fibrils were present; therefore, collagen was not detectable using DIC microscopy. Quantitative analysis was performed by using a Fourier Transform algorithm to determine the percent of image content aligned at each radial angle within the image [28]. Random collagen fibrils showed no preferential alignment, whereas a peak in alignment near 0° was generally observed for the aligned collagen substrates (Figure 1C).

**Figure 2:**
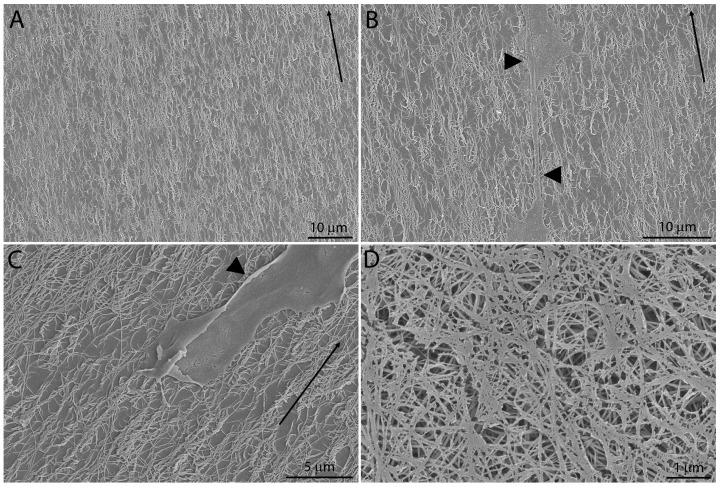
Scanning electron microscopy (SEM) images of collagen substrates. (**A**) Aligned collagen fibrils on a substrate without a freeze injury; (**B**) Aligned collagen fibrils and corneal keratocytes (arrowheads) outside the region of the freeze injury, after four days of culture in media containing PDGF; (**C**) Aligned collagen fibrils and a cell (arrowhead) within the region of the freeze injury, after four days of culture in media containing PDGF; (**D**) Collagen fibrils in an unaligned substrate. Arrows indicate direction of flow used during collagen deposition in aligned substrates.

Scanning electron microscopy (SEM) imaging was used to view collagen fibrils at a higher magnification. The microfluidics channels deposited collagen fibrils that were generally aligned parallel to the direction of flow (Figure 2A); however higher magnification revealed that fibrils were wavy and interwoven (Figure 2B). At higher magnification, some collagen fibrils appeared to have a twisted, rope-like structure (not shown), similar to what has been shown previously by Saeidi and associates [34]. Importantly, there was no apparent difference detected in collagen structure between the non-injured (Figure 2A,B) and freeze-injured (Figure 2C) regions. Cell processes extended on top of collagen fibrils in alignment with the collagen, and no obvious disruption of the collagen architecture was observed surrounding cells (Figure 2B,C arrowheads). Collagen in unaligned substrates was thicker, more interwoven and appeared randomly aligned (Figure 2D).

### 2.2. Effect of Substrate Alignment on Corneal Keratocyte Patterning

**Figure 3:**
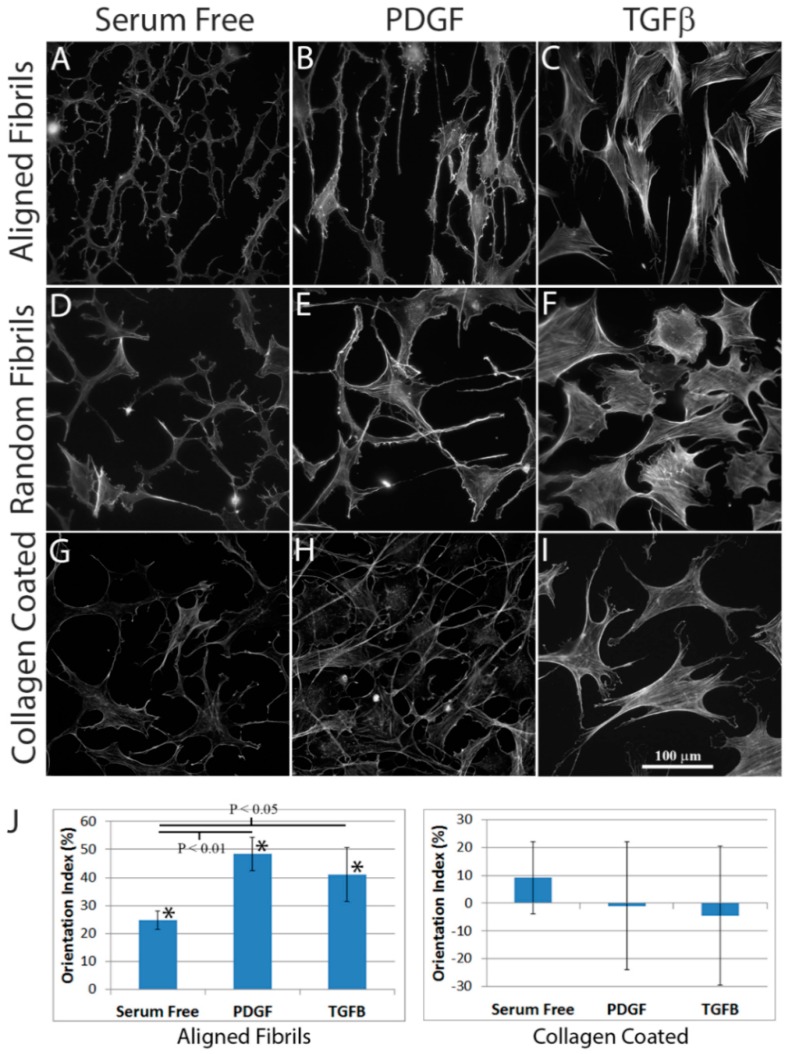
Influence of substrate topography on corneal keratocyte differentiation and alignment. (**A**–**I**) F-actin labeling of keratocytes plated on aligned fibrillar collagen, random fibrillar collagen, or unpolymerized collagen-coated substrates, four days after incubation in serum-free media, or serum-free media supplemented with PDGF or TGFβ. Collagen is patterned vertically on the aligned substrates; (**J**) Positive orientation index (OI) values were found for cells on aligned substrates under all three culture conditions (*, *p* < 0.001), indicating co-alignment of cells with the direction of the aligned collagen fibrils (left panel). However, cells cultured in the presence of PDGF or TGFβ had higher orientation indices on aligned substrates than cells cultured in basal serum free media (One way analysis of variance using images from at 4 independent experiments per condition). Cells cultured on collagen-coated dishes had mean OI values close to zero, indicating random cell alignment (right panel).

Corneal keratocytes were plated on aligned fibrillar collagen, random fibrillar collagen, or unpolymerized collagen-coated substrates. A freeze injury was then made in the center of the substrate. After 4 days of incubation in basal (serum-free) media, or basal media supplemented with PDGF or TGFβ, the constructs were fixed and labeled for F-actin (Figure 3). Keratocytes in serum-free media had a dendritic morphology, and did not express stress fibers on any of the substrates. Keratocytes on aligned collagen generally co-aligned with the collagen fibrils (Figure 3A), whereas keratocytes plated on unaligned collagen (Figure 3D) or unpolymerized collagen-coated dishes (Figure 3G) were randomly oriented. Cells in PDGF-containing media were narrow, elongated, and did not express stress fibers on any substrates studied. Cells in PDGF were highly co-aligned with the collagen fibrils on aligned substrates (Figure 3B), but were randomly oriented on unaligned collagen (Figure 3E) or collagen-coated substrates (Figure 3H). Cells in TGFβ expressed prominent stress fibers that were generally co-aligned with the collagen fibrils on aligned collagen substrates (Figure 3C), but were randomly oriented on unaligned collagen (Figure 3F) or collagen-coated substrates (Figure 3I). Overall, these findings were consistent with previous studies showing cell alignment with ridges on patterned polyurethane substrates [35]. An important advantage of aligned collagen substrates is that Type I collagen fibrils are the main component of the corneal stroma, thus studying their interactions with collagen fibrils may be more physiological. However, a limitation in the current model is that unlike polyeurathane substrates and the native corneal stromal lamellae, collagen fibrils on the aligned substrates were somewhat wavy and interwoven. However, this could potentially be improved with further optimization of the microfluidics device and flow parameters.

To assess whether cells cultured in TGFβ media transformed into myofibroblasts, α-SMA labeling was used (Figure S1). In both fibrillar collagen substrates and unpolymerized collagen-coated substrates, α-SMA was expressed in 60–70% of cells, and was colocalized with F-actin labeled stress fibers. Previous findings using a rabbit model indicate that following transcorneal freeze-injury (FI), keratocytes transform into fibroblasts and migrate into the wound site parallel to the orientation of the stromal lamellae [27]. Interestingly, cells do not transform into myofibroblasts or contain stress fibers, suggesting a possible phenotype dependence on mechanical and/or biochemical cues from the stromal ECM. Earlier in vitro studies using aligned electrospun Type I collagen fibrils revealed a reduction in myofibroblast transformation of corneal fibroblasts after stimulation with TGFβ compared to fibroblasts seeded on unaligned fibrils, collagen gels, and tissue culture plates [36]. Studies by Myrna and co-workers showed that myofibroblast transformation also depended on the “pitch” (groove + ridge width) of the aligned topography within substrates, with a higher “pitch” reducing myofibroblast transformation [20]. Overall, it is likely that a combination of ECM topography, stiffness, protein composition, and structure (fibrillar vs. monomeric) integrate to modulate corneal keratocyte behavior, patterning, and phenotype [37].

To quantify the extent of cell alignment, orientation indexes (OI) were calculated. The OI measures the extent of alignment of image content with respect to a reference angle, with 0% indicating no alignment, 100% indicating parallel alignment, and −100% indicating perpendicular alignment. An angle of 90° was used as the reference angle, since collagen fibrils were patterned vertically in these images. Note that because of the differences in collagen thickness and fibril density on random substrates (Figure 2D), experiments for quantitative analysis were only performed using unpolymerized collagen-coated and aligned collagen substrates. As shown in Figure 3J (left panel), positive OI values were found for cells on aligned substrates under all 3 culture conditions, indicating significant co-alignment with the direction of collagen fibrils. In addition, treatment with PDGF or TGFβ produced higher orientation indices on aligned substrates, as compared to cells cultured in serum free basal media. Cells cultured on collagen-coated dishes had mean OI values close to zero, indicating random cell alignment (Figure 3J, right panel).

**Figure 4:**
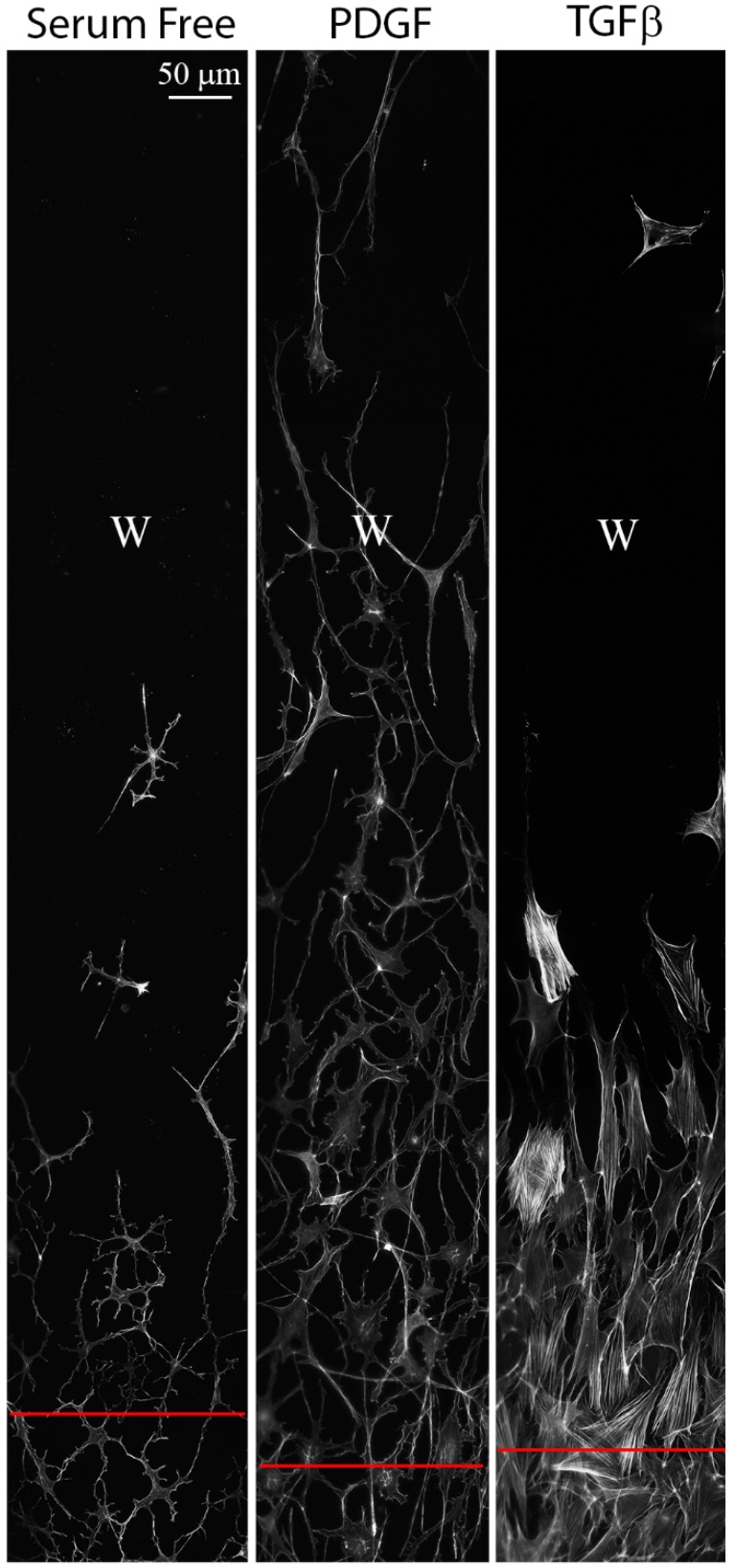
Cell patterning during wound repopulation following freeze injury on aligned collagen substrates. Each panel is a montage of F-actin images collected four days after injury. “W” marks the center of the original wound area, and red lines mark the approximate location of the original wound edge. The aligned collagen fibrils are patterned vertically.

Montages showing the overall pattern of cell spreading and migration on aligned collagen substrates are shown in Figure 4. In general, wound areas were found to completely repopulate with cells plated on aligned substrates when cultured with PDGF, but not when cultured in serum free media or TGFβ. Migrating cells tended to remain interconnected in PDGF whereas individual cell migration was often observed in TGFβ. In contrast, keratocytes failed to fully repopulate the wound site on unpolymerized collagen-coated substrates regardless of the culture conditions (not shown). These findings are consistent with previous studies that indicate cells migrate faster while on aligned substrates [35]. Interestingly, cells were generally aligned vertically all the way across the wound area, suggesting that they did not migrate in as much from the sides, i.e., perpendicular to the collagen fibrils (Figure S2, W indicates center of wound and red line marks original wound edge). Overall, the novel FI model allows changes in cell morphology and cytoskeletal organization during wound repopulation to be evaluated on a patterned fibrillar substrate.

### 2.3. Time-Lapse Imaging of Cell-Collagen Interactions

**Figure 5:**
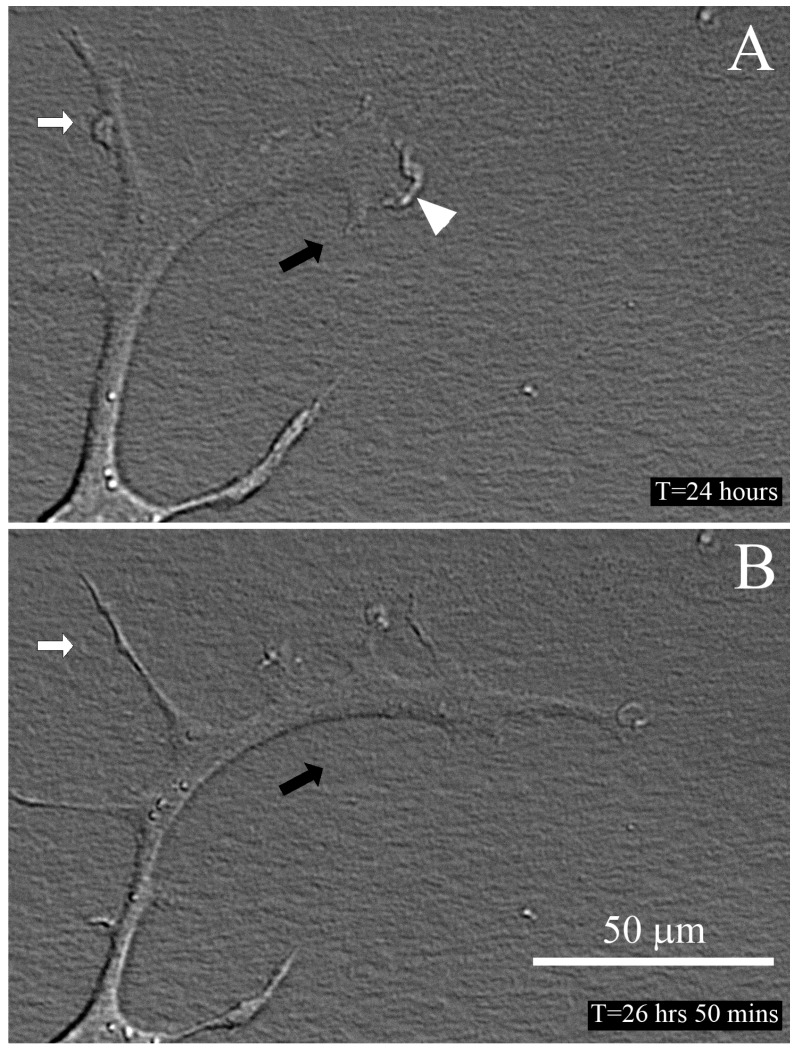
Live cell DIC imaging of cell-collagen interactions. Still images from a time-lapse recording of a cell migrating on an aligned collagen substrate under serum free culture conditions, at the leading edge of a freeze injury (wounded area is to the right). The collagen fibrils are aligned horizontally. Membrane ruffling was observed at the leading edge of the cell (**A**, arrowhead), and the main direction of movement was in parallel with the aligned collagen (compare cell position with respect to black reference arrows in **A**,**B**). Interestingly, several thin processes extended from the cell body both parallel and perpendicular (white arrows) to the collagen fibrils, and some of these process persisted over time. Time is relative to when the freeze injury was made. See also Video S1.

In order to visualize the dynamic interactions between cells and collagen fibrils, high magnification time-lapse DIC imaging was used [38,39]. An example of cell motility under serum free culture conditions is shown in Figure 5 and Video S1. Membrane ruffling was observed at the leading edge of the cell, and the main direction of movement was in parallel with the aligned collagen (horizontal). Interestingly, several thin processes extended from the cell body both parallel and perpendicular (white arrows) to the collagen fibrils, and some of these processes persisted over time.

**Figure 6:**
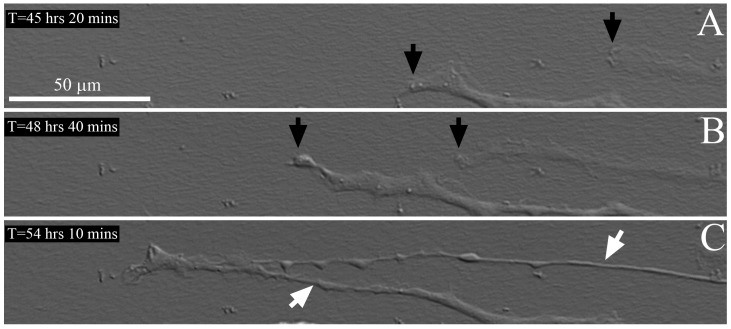
Live cell DIC imaging of cell-collagen interactions. Still images from a time-lapse recording of cells migrating on an aligned collagen substrate under serum free + PGDF culture conditions, at the leading edge of a freeze injury (wounded area is to the left). The collagen fibrils are aligned horizontally. (**A**,**B**) The leading edge of two cells is observed (black arrows). The leading edges ruffle as the cells extend, and the cells migrate parallel to the alignment of the collagen; (**C**) Over time, the two cells interconnect and move together at the leading edge. As the cells continue to elongate, the processes became much thinner. Time is relative to when the freeze injury was made. See also Video S2.

Time-lapse imaging of two cells migrating on an aligned collagen substrate under serum free + PGDF culture conditions is shown in Figure 6 and Video S2. The leading edges ruffle as the cells extend, and the cells migrate parallel to the alignment of the collagen. Over time, the two cells interconnect and move together at the leading edge. As the cells continued to elongate, the processes became much thinner. In general, cells were more elongated in PDGF and had fewer processes along the sides of the cell body as compared to cells in serum free media. When there was extension of processes on the sides of the cells during migration, the activity of these processes was generally limited, which is displayed in Video S3. Some processes begin to spread from the main body of the cell but do not persist, possibly because they are not in alignment with the collagen. When a gap in the collagen is encountered at the leading edge, the cell reaches across it to find another area with collagen fibrils (arrow in Video S3).

**Figure 7:**
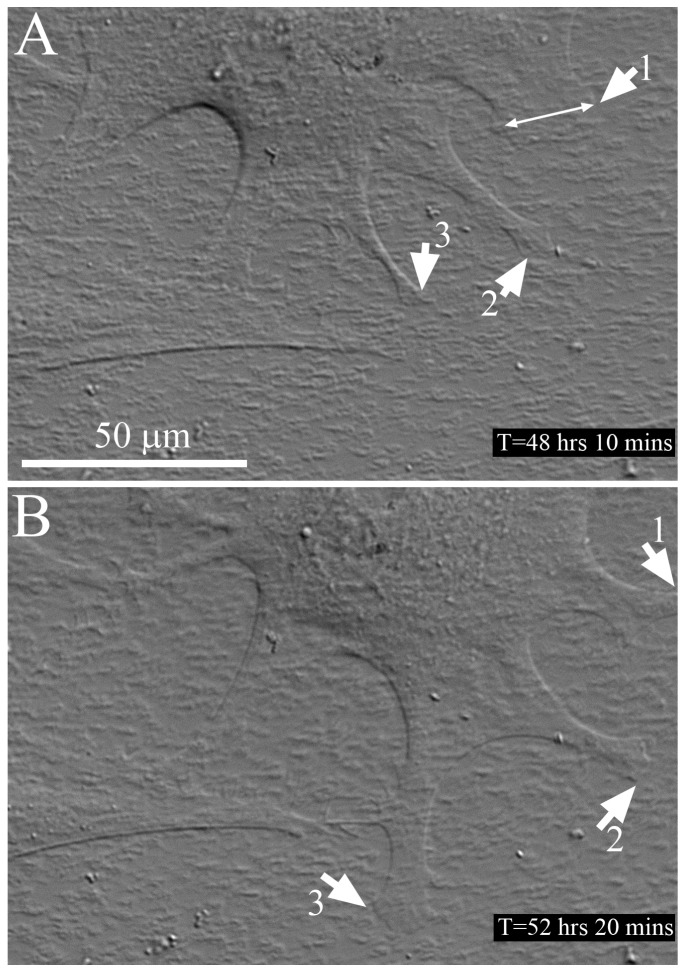
Live cell DIC imaging of cell-collagen interactions. Still images from a time-lapse recording of cell migrating on an aligned collagen substrate under serum free + TGFβ culture conditions, near the leading edge of a freeze injury (wounded area is to the right). The collagen fibrils are aligned horizontally. (**A**) Note that the cell has a circular morphology with processes extending in all directions. Broader processes are often observed (double arrow); (**B**) Processes 1 and 2 have extended in parallel with the collagen fibrils, whereas process 3 extended perpendicular to the fibril alignment. Time is relative to when the freeze injury was made. See also Video S4.

Time-lapse imaging of cells migrating on an aligned collagen substrate under serum free + TGFβ culture conditions is shown in Figure 7 and Video S4. In TGFβ, cells had a more circular morphology with processes extending in all directions. Broader processes were often observed (double arrow), as compared to serum free and PDGF. Note that some processes (1 and 2) extended in parallel with the collagen fibrils, whereas another (3) extended perpendicular to the fibril alignment. Despite the fact that these cells had stress fibers and were likely exerting significant forces on the collagen ECM, no movement or separation of the collagen fibrils from the substrate was observed during time-lapse imaging.

Other time-lapse videos provided additional insights into cellular interactions with the aligned substrates. In Video S5, rapid retraction of a cell process at the trailing edge is observed, suggesting rupture of cell-ECM adhesion. Interestingly, the cell re-extends along the same path, suggesting that a track of cell-deposited protein may have been left behind. Consistent with this observation, corneal fibroblasts have been shown to form interconnected aligned chains during intrastromal cell migration in vivo, and in vitro studies in 3-D fibrin matrices suggest that they can follow fibronectin tracks left behind by other cells [27,40]. Another example, taken at the edge of the aligned fibril substrate (Video S6), shows the difference in cell behavior between glass and aligned collagen. When on the glass (first white arrow), the cell migrated vertically. Once the cell reached the collagen coated region (second white arrow), processes began to turn in alignment with the collagen (horizontal), and over time the cell reoriented to become more parallel with the collagen fibrils. By incorporating live cell labeling of focal adhesions or cytoskeletal proteins, additional insights into the mechanical interactions between cells and aligned collagen fibrils are possible with this model [41].

## 3. Materials and Methods

### 3.1. Fabrication of Microfluidics Device

Microfluidic devices were fabricated in polydimethylsiloxane (PDMS) as previously described [42]. Briefly, a standard negative photolithography procedure was utilized to fabricate a straight channel photoresist template (height = 60 µm, width = 1500 µm, length = 22,000 µm) in KMPR 1050 photoresist (Micr Chem, Westborough, MA, USA). Next, the PDMS prepolymer and crosslinker (Sylgard 184 Elastomer kit, Dow Corning, Michigan, MI, USA) were mixed in a 10:1 weight ratio, poured over the photoresist structure, and then placed under vacuum for one hour to remove air bubbles. Plastic elbows (Value Plastics Inc., Fort Collins, CO, USA) were placed at the outlet port of the device and the PDMS was cured in an oven for at least 1 h at 80 °C.

To complete the microfluidic device, PDMS stamps were attached to hydrophobic glass surfaces. Glass coverslips (#1.5) were made hydrophobic by soaking in a 28.8% Nitric Acid Solution for one hour, oven-dried, washed 3 times with Millipore water, and placed into a 1% Aquasil solution (Thermo Fisher Scientific, Waltham, MA, USA) for 15 s. To ensure a tight seal with the glass coverslips during collagen infusion through the channel, the PDMS stamps were exposed to an air plasma in a Plasma Cleaner (Model PDC-32G; Harrick; Ithaca, NY, USA) for one minute at the high power setting.

### 3.2. Fabrication of Collagen Substrates

On ice, 8 parts bovine Type I collagen solution (3.0 mg/mL, PureCol, Advanced BioMatrix, San Diego, CA, USA) was mixed with 1 part 10×minimal essential media (MEM) and 1 part NaOH to adjust the pH to physiological levels for collagen polymerization. One× MEM was also added to adjust the final concentration of collagen to 1.6 mg/mL. The solution was drawn into a 1 mL syringe connected to tubing (Silastic, Dow Corning), ensuring no bubbles. Immediately after, the tubing was connected to the PDMS device, and collagen solution was gently injected into the channel (Figure 1). Once collagen had reached the outlet of the channel within the device, the syringe was quickly placed into a syringe pump (Harvard Apparatus, Holliston, MA, USA), which was set at a flow rate of 7.5 μL/min, which corresponds to a shear rate of 150 s^−1^. The device was placed on a hot plate set at 37 °C to initiate collagen polymerization. After 30 min, the PDMS was removed from the glass and the newly formed collagen was rinsed carefully with Millipore water. Slides with aligned collagen were left to dry at room temperature. After the channel dried, a 30-mm diameter PDMS ring (cleaned in a PlasmaFlo chamber with high RF, 300 mTorr for 1 min) was inserted on top of the slide, creating a well for the cell suspension.

To produce randomly oriented fibrillar collagen substrates, a PDMS ring was placed on top of the Aquasil coated glass slide, and neutralized cold collagen solution (1.6 mg/mL) was poured into the well to create a thin layer. The construct was then incubated for 30 min at 37 °C for polymerization.

To produce standard, unpolymerized collagen-coated substrates, a 50 μg/mL neutralized solution of bovine Type I collagen solution was dispensed into a glass bottom 30-mm diameter poly-d-lysine coated MatTek dish and placed in a humidified incubator (37 °C, 5% CO_2_) for 30 min, as previously described [43].

All dishes were rinsed twice with Dulbecco’s modified Eagle’s medium (DMEM; Sigma-Aldrich, St. Louis, MO, USA) supplemented with 1% RPMI vitamin mix (Sigma-Aldrich, St. Louis, MO, USA), 100 µM nonessential amino acids (Invitrogen), 100 µg/mL ascorbic acid, and 1% penicillin/streptomycin (Invitrogen) prior to adding cells.

### 3.3. Cell Culture

To study cell behavior on the collagen substrates, primary rabbit corneal keratocytes (NRK) cells were extracted from New Zealand White Rabbit eyes (Pel-Freez, Rogers, AR, USA), as previously described [44]. Cells were cultured in basal media consisting of Dulbecco’s modified Eagle’s medium (DMEM; Sigma-Aldrich, St. Louis, MO, USA) supplemented with 1% RPMI vitamin mix (Sigma-Aldrich, St. Louis, MO, USA), 100 µM nonessential amino acids (Invitrogen), 100 µg/mL ascorbic acid, and 1% penicillin/streptomycin (Invitrogen) [45,46]. First passage cells cultured for 4–5 days were used in all experiments. 

### 3.4. Freeze Injury (FI)

To study the effects of topography on cell spreading and migration, a FI model was used (Figure S3). For FI experiments, cells were seeded at a density of 50,000 cells/mL (2 mL per well) with either basal serum free media (described above), or basal media supplemented with PDGF BB (50 ng/mL) or transofrming growth factor beta 1 (TGFβ) (5 ng/mL) for 24 h. To create the FI, media was removed, and a 1 mm stainless steel probe was dipped in liquid nitrogen for 10 s. In the center of the dish (for random collagen and collagen-coated) and in the middle of the channel (for aligned collagen), the probe was placed on the back (glass) side of the construct for 10 s immediately after dipping in liquid nitrogen. Any detached or partially detached cells were then washed away with DMEM serum-free media, and the slide was checked to verify cell detachment in the wound area. Following injury, basal media or basal media supplemented with PDGF BB or TGFβ was re-added, and the cells were left for 4 additional days in a humidified incubator (37 °C, 5% CO_2_) to allow for differentiation and migration.

### 3.5. Immunocytochemistry

For labeling of F-actin, at the end of each experiment, cells were fixed using a 3% paraformaldehyde solution for 10 min, washed two times for 10 min each, permeabilized using 0.5% Triton X-100 in phosphate buffered saline (PBS) for 15 min, washed once for 10 min, and incubated with Alexa Fluor 488 Phalloidin (1:100, Molecular Probes, Eugene, OR, USA) for 1 h at 37 °C. For double labeling of alpha-smooth muscle actin (α-SMA) and F-actin, samples were blocked with 1% bovine serum albumin (BSA) fraction V in PBS for 1 h following permeabilization. Cells were then washed three times, 20 min per wash, and then incubated with primary mouse anti-human α-SMA antibody (1:600, Sigma, St. Louis, MO, USA) for 2 h at 37 °C. Cells were then washed three times, 20 min per wash, and then incubated with FITC conjugated goat anti-mouse secondary antibody (1:200) and Alexa Fluor 546 Phalloidin (1:150) at 37 °C for 1 h. The collagen and cells were imaged using a Nikon TE300 inverted microscope (TE300; Nikon, Tokyo, Japan) with differential interference contrast (DIC), phase, and fluorescent capabilities using a 40× water immersion objective. 

### 3.6. Scanning Electron Microscopy (SEM)

For SEM imaging, samples were fixed with a 2.5% glutaraldehyde in 0.1 M cacodylate buffer solution for 2 h at room temperature. Samples were then rinsed in 0.1 M cacodylate buffer three times, and fixed again with 2% osmium in 0.1 M Na cacodylate buffer. After rinsing five times with Millipore water, samples were dehydrated using an increasing concentration of ethanol (50–100%). Following dehydration, samples were placed into a critical point dryer, and sputter-coated. Collagen fibrils were viewed using a field emission SEM (Zeiss Sigma, Jena, Germany) at 3 kV.

### 3.7. Orientation Index

Cell alignment was quantified using an in-house MATLAB program (MathWorks, Natick, MA, USA), which uses a Fourier Transform algorithm to determine the percent of image content aligned at each radial angle within an image (I(∅)) [28]. The degree of cell alignment was calculated using a previously described orientation index (OI), using the following equation:

$$OI\;\left(\theta \right)=\left\{2<\cos^{2}\theta >-1\right\}100\%$$

where,

$$\cos^{2}\theta =\frac{\int_{-90}^{90}I\left(\emptyset \right)\left\{\cos^{2}\left(\emptyset -\theta \right)\right\}d\emptyset }{\int_{-90}^{90}I\left(\emptyset \right)d\emptyset }$$

and θ = 0° is the x axis within the image [47]. In this study, the angle selected was 90°, which corresponds to the direction of the aligned collagen fibrils in the samples analyzed (i.e., direction of flow during deposition). For cells completely aligned with collagen, the OI is equal to 100%; whereas for cells completely perpendicular to the collagen fibrils, the OI is equal to −100%, and for randomly aligned fibrils the OI is equal to 0%. 

### 3.8. Statistical Analysis

SigmaPlot (version 12.5; Systat Software, Inc., San Jose, CA, USA) was used for statistical analysis. One way analysis of variance (ANOVA) was used for comparing OI values under different culture conditions. Post-hoc analysis using the Holm-Sidak method was used for comparisons between groups.

### 3.9. Time-Lapse Imaging

In a subset of samples, time-lapse DIC imaging was conducted beginning 24 h after freeze injury. A Nikon Eclipse inverted microscope (Nikon, Tokyo, Japan) equipped with an environmental chamber (In Vivo Scientific, Saint Louis, MO, USA) was used, as previously described [48,49]. Z-series of DIC images were taken at multiple regions near the leading edge of the wound at 10 min intervals using a 60× oil immersion objective. Imaging was carried out for up to 72 h. To create movies of cell movements, a single plane from the z-series was selected at each time point, so that the cells were in focus for the entire sequence. MetaMorph software was used to generate movies of cell movements.

## 4. Conclusions

Previous studies have demonstrated that ECM stiffness, topography, dimensionality, and protein composition all have the potential to modulate corneal keratocyte differentiation, contractility, and patterning. Disentangling the relationships between these cues requires sophisticated multifactorial culture platforms in which these parameters can be modulated independently, which is not possible using in vivo models or organ culture systems. A better understanding of how keratocytes integrate combinations of cues is critical for developing novel therapies to reduce or reverse corneal scarring, or to guide tissue engineering approaches to developing stromal tissue replacements. The microfluidics model presented allows assessment of corneal keratocyte differentiation and motility in response to topographic signaling (aligned collagen), changes in the growth factor environment, and wounding (freeze injury). Microfluidics is a flexible platform, and with continued development, such as the addition of other protein patterns or variations in substrate elasticity, this experimental model could be an important tool for accessing how the interplay between biophysical and biochemical signals regulate corneal keratocyte differentiation and mechanical behavior.

## Supplementary Materials

The following are available online at http://www.mdpi.com/2079-4983/9/4/54/s1, Figure S1, Myofibroblast transformation in response to TGFβ, Figure S2, Cell patterning across an aligned substrate, Figure S3, Schematic of freeze injury model, Video S1, Cell migrating on an aligned collagen substrate in serum free media, Video S2, Cells migrating on an aligned collagen substrate in PDGF, Video S3, Rapidly migrating cell on an aligned collagen substrate in PDGF, Video S4, Cell migrating on an aligned collagen substrate in TGFβ, Video S5, Trailing edge of migrating cell on an aligned collagen substrate in PDGF, Video S6, Cell migrating across the glass-collagen interface in PDGF.

## References

[1] Perez-Gomez I., Efron N. (2003). Change to corneal morphology after refractive surgery (myopic laser in situ keratomileusis) as viewed with a confocal microscope. Optom. Vis. Sci..

[2] Chakravarti S., Petroll W.M., Hassell J., Jester J.V., Lass J.H., Paul J., Birk D.E. (2000). Corneal opacity in lumican-null mice: Defects in collagen fibril structure and packing in the posterior stroma. Investig. Ophthalmol. Vis. Sci..

[3] Funderburgh J.L., Mann M.M., Funderburgh M.L. (2003). Keratocyte phenotype mediates proteoglycan structure: A role for fibroblasts in corneal fibrosis. J. Biol. Chem..

[4] Hassell J.R., Birk D.E. (2010). The molecular basis of corneal transparency. Exp. Eye Res..

[5] Engler A.J., Sen S., Sweeney H.L., Discher D.E. (2006). Matrix elasticity directs stem cell lineage specification. Cell.

[6] Krieg M., Arboleda-Estudillo Y., Puech P.-H., Kafer J., Graner F., Muller D.J., Heisenberg C.-P. (2008). Tensile forces govern germ-layer organization in zebrafish. Nat. Cell Biol..

[7] Stopak D., Wessels N.K., Harris A.K. (1985). Morphogenetic rearrangement of injected collagen in developing chicken limb buds. Proc. Natl. Acad. Sci. USA.

[8] Bard J.B.L., Higginson K. (1977). Fibroblast-collagen interactions in the formation of the secondary stroma of the chick cornea. J. Cell Biol..

[9] Garana R.M., Petroll W.M., Chen W.T., Herman I.M., Barry P., Andrews H.D., Cavanagh H.D., Jester J.V. (1992). Radial keratotomy II: The role of the myofibroblast in corneal wound contraction. Investig. Ophthalmol. Vis. Sci..

[10] Moller-Pedersen T., Li H., Petroll W.M., Cavanagh H.D., Jester J.V. (1998). Confocal microscopic characterization of wound repair after photorefractive keratectomy using in vivo confocal microscopy. Investig. Ophthalmol. Vis. Sci..

[11] Stramer B.M., Zieske J.D., Jung J.-C., Austin J.S., Fini M.E. (2003). Molecular mechanisms controlling the fibrotic repair phenotype in cornea: Implications for surgical outcomes. Investig. Ophthalmol. Vis. Sci..

[12] Varner V.D., Nelson C.M. (2014). Toward the directed self-assembly of engineered tissues. Annu. Rev. Chem. Biomol. Eng..

[13] Gouveia R.M., Gonzalez-Andrades E., Cardona J.C., Gonzalez-Gallardo C., Ionescu A.M., Garzon I., Alaminos M., Gonzalez-Andrades M., Connon C.J. (2017). Controlling the 3D architecture of self-lifting auto-generated tissue equivalents (slates) for optimized corneal graft composition and stability. Biomaterials.

[14] Kivanany P.B., Grose K.C., Tippani M., Su S., Petroll W.M. (2018). Assessment of corneal stromal remodeling and regeneration after photorefractive keratectomy. Sci Rep..

[15] Cintron C., Kublin C.L. (1977). Regeneration of corneal tissue. Dev. Biol..

[16] Etheredge L., Kane B.P., Hassell J.R. (2009). The effect of growth factor signaling on keratocytes in vitro and its relationship to the phases of stromal wound repair. Investig. Ophthalmol. Vis. Sci..

[17] Ghibaudo M., Trichet L., Le Digabel J., Richert A., Hersen P., Ladoux B. (2009). Substrate topography induces a crossover from 2d to 3d behavior in fibroblast migration. Biophys. J..

[18] Teixeira A.I., Nealey P.F., Murphy C.J. (2004). Responses of human keratocytes to micro- and nanostructured substrates. J. Biomed. Mater. Res. A.

[19] Teixeira A.I., Abrams G.A., Bertics P.J., Murphy C.J., Nealey P.F. (2003). Epithelial contact guidance on well-defined micro- and nanostructured substrates. J. Cell Sci..

[20] Myrna K.E., Mendonsa R., Russell P., Pot S.A., Liliensiek S.J., Jester J.V., Nealey P.F., Brown D., Murphy C.J. (2012). Substratum topography modulates corneal fibroblast to myofibroblast transformation. Investig. Ophthalmol. Vis. Sci..

[21] Guillemette M.D., Cui B., Roy E., Gauvin R., Giasson C.J., Esch M.B., Carrier P., Deschambeault A., Dumoulin M., Toner M. (2009). Surface topography induces 3D self-orientation of cells and extracellular matrix resulting in improved tissue function. Integr. Biol..

[22] Saeidi N., Guo X., Hutcheon A.E., Sander E.A., Bale S.S., Melotti S.A., Zieske J.D., Trinkaus-Randall V., Ruberti J.W. (2012). Disorganized collagen scaffold interferes with fibroblast mediated deposition of organized extracellular matrix in vitro. Biotechnol. Bioeng..

[23] Guo X.Q., Hutcheon A.E., Melotti S.A., Zieske J.D., Trinkaus-Randall V., Ruberti J.W. (2007). Morphologic characterization of organized extracellular matrix deposition by ascorbic acid-stimulated human corneal fibroblasts. Investig. Ophthalmol. Vis. Sci..

[24] Karamichos D., Guo X.Q., Hutcheon A.E., Zieske J.D. (2010). Human corneal fibrosis: An in vitro model. Investig. Ophthalmol. Vis. Sci..

[25] Ren R., Hutcheon A.E.K., Guo X.Q., Saeidi N., Melotti S.A., Ruberti J.W., Zieske J.D., Trinkaus-Randall V. (2008). Human primary corneal fibroblasts synthesize and deposit proteoglycans in long-term cultures. Dev. Dyn..

[26] Karamichos D., Funderburgh M.L., Hutcheon A.E., Zieske J.D., Du Y., Wu J., Funderburgh J.L. (2014). A role for topographic cues in the organization of collagenous matrix by corneal fibroblasts and stem cells. PLoS ONE.

[27] Petroll W.M., Kivanany P.B., Hagenasr D., Graham E.K. (2015). Corneal fibroblast migration patterns during intrastromal wound healing correlate with ecm structure and alignment. Investig. Ophthalmol. Vis. Sci..

[28] Kivanany P.B., Grose K.C., Petroll W.M. (2016). Temporal and spatial analysis of stromal cell and extracellular matrix patterning following lamellar keratectomy. Exp. Eye Res..

[29] Flynn B.P., Bhole A.P., Saeidi N., Liles M., Dimarzio C.A., Ruberti J.W. (2010). Mechanical strain stabilizes reconstituted collagen fibrils against enzymatic degradation by mammalian collagenase matrix metalloproteinase 8 (mmp-8). PLoS ONE.

[30] Bhole A.P., Flynn B.P., Liles M., Saeidi N., Dimarzio C.A., Ruberti J.W. (2009). Mechanical strain enhances survivability of collagen micronetworks in the presence of collagenase: Implications for load-bearing matrix growth and stability. Philos. Trans. A Math. Phys. Eng. Sci..

[31] Grinnell F., Petroll W.M. (2010). Cell motility and mechanics in three-dimensional collagen matrices. Annu. Rev. Cell Dev. Biol..

[32] Karamichos D., Brown R.A., Mudera V. (2007). Collagen stiffness regulates cellular contraction and matrix remodeling gene expresssion. J. Biomed. Mater. Res. A.

[33] Lanfer B., Freudenberg U., Zimmermann R., Stamov D., Korber V., Werner C. (2008). Aligned fibrillar collagen matrices obtained by shear flow deposition. Biomaterials.

[34] Saeidi N., Sander E.A., Ruberti J.W. (2009). Dynamic shear-influenced collagen self-assembly. Biomaterials.

[35] Pot S.A., Liliensiek S.J., Myrna K.E., Bentley E., Jester J.V., Nealey P.F., Murphy C.J. (2010). Nanoscale topography–induced modulation of fundamental cell behaviors of rabbit corneal keratocytes, fibroblasts, and myofibroblasts. Investig. Ophthalmol. Vis. Sci..

[36] Phu D., Wray L.S., Warren R.V., Haskell R.C., Orwin E.J. (2011). Effect of substrate composition and alignment on corneal cell phenotype. Tissue Eng. Part. A.

[37] Dreier B., Thomasy S.M., Mendonsa R., Raghunathan V.K., Russell P., Murphy C.J. (2013). Substratum compliance modulates corneal fibroblast to myofibroblast transformation. Investig. Ophthalmol. Vis. Sci..

[38] Petroll W.M., Ma L. (2003). Direct, dynamic assessment of cell-matrix interactions inside fibrillar collagen lattices. Cell Motil. Cytoskel..

[39] Petroll W.M., Ma L., Kim A., Ly L., Vishwanath M. (2008). Dynamic assessment of fibroblast mechanical activity during rac-induced cell spreading in 3-D culture. J. Cell. Physiol..

[40] Miron-Mendoza M., Graham E., Manohar S., Petroll W.M. (2017). Fibroblast-fibronectin patterning and network formation in 3D fibrin matrices. Matrix Biol..

[41] Petroll W.M., Ma L., Jester J.V. (2003). Direct correlation of collagen matrix deformation with focal adhesion dynamics in living corneal fibroblasts. J. Cell Sci..

[42] Shimp E.A., Alsmadi N.Z., Cheng T., Lam K.H., Lewis C.S., Schmidtke D.W. (2016). Effects of shear on p-selectin deposition in microfluidic channels. Biomicrofluidics.

[43] Miron-Mendoza M., Graham E., Kivanany P., Quiring J., Petroll W.M. (2015). The role of thrombin and cell contractility in regulating clustering and collective migration of corneal fibroblasts in different ecm environments. Investig. Ophthalmol. Vis. Sci..

[44] Lakshman N., Kim A., Petroll W.M. (2010). Characterization of corneal keratocyte morphology and mechanical activity within 3-D collagen matrices. Exp. Eye Res..

[45] Jester J.V., Barry P.A., Lind G.J., Petroll W.M., Garana R., Cavanagh H.D. (1994). Corneal keratocytes: In situ and in vitro organization of cytoskeletal contractile proteins. Investig. Ophthalmol. Vis. Sci..

[46] Lakshman N., Petroll W.M. (2012). Growth factor regulation of corneal keratocyte mechanical phenotypes in 3-D collagen matrices. Investig. Ophthalmol. Vis. Sci..

[47] Kim A., Lakshman N., Petroll W.M. (2006). Quantitative assessment of local collagen matrix remodeling in 3-D culture: The role of Rho kinase. Exp. Cell Res..

[48] Zhou C., Petroll W.M. (2010). Rho kinase regulation of fibroblast migratory mechanics in fibrillar collagen matrices. Cell. Mol. Bioeng..

[49] Zhou C., Petroll W.M. (2014). Mmp regulation of corneal keratocyte motility and mechanics in 3-D collagen matrices. Exp. Eye Res..

