# Supplementary figures and images for: An In Vitro Model for Assessing Corneal Keratocyte Spreading and Migration on Aligned Fibrillar Collagen
Source: J Funct Biomater. 2018 Sep 21;9(4):54. doi: 10.3390/jfb9040054 (PMC6306816; doi:10.3390/jfb9040054)

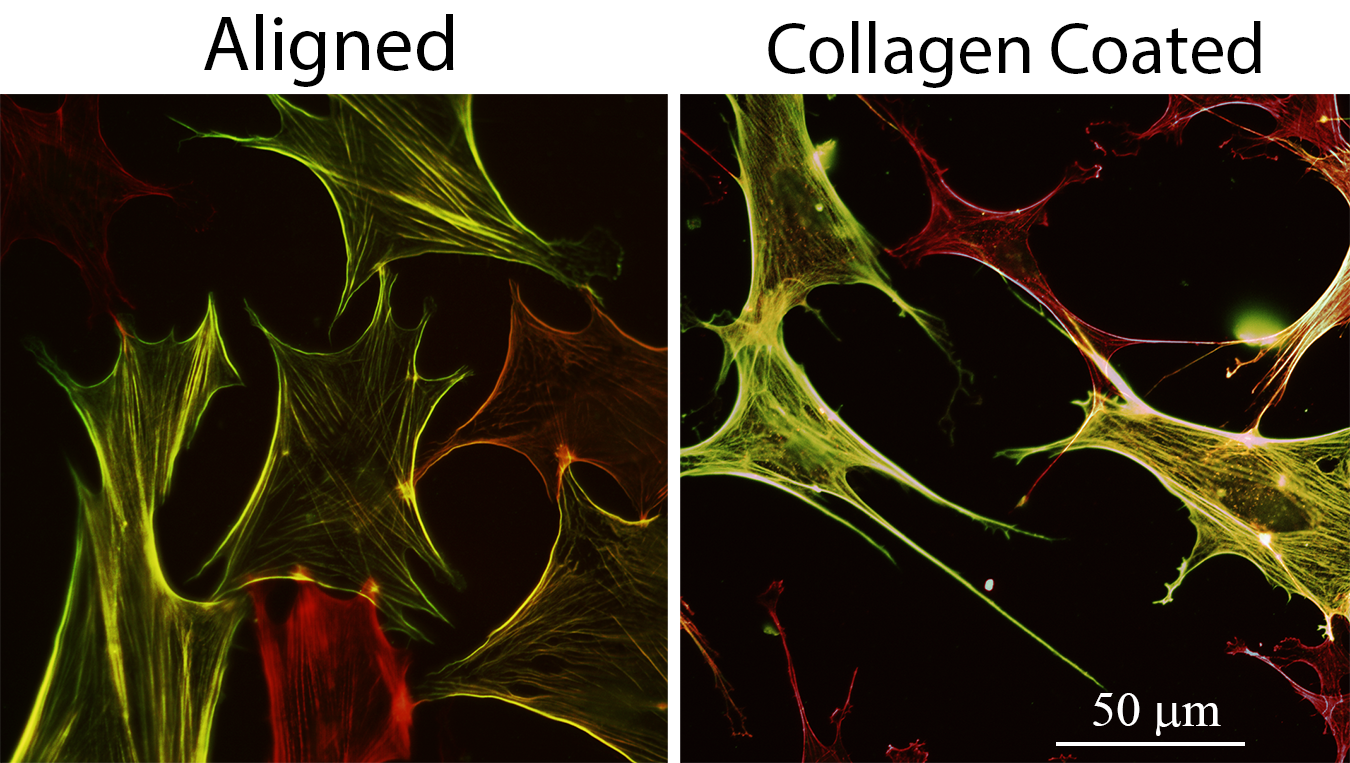

Supplement: Supplementary file 1 [file jfb-09-00054-s001.zip › SuppMaterial/Fig S1.tif]

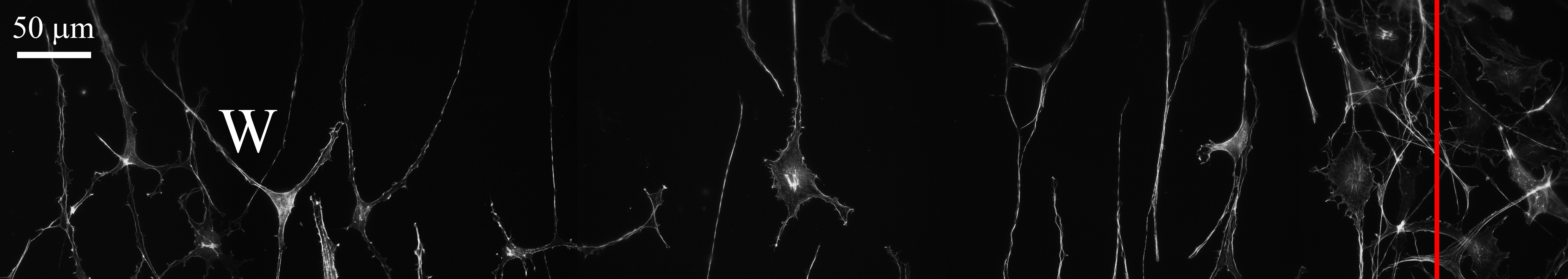

Supplement: Supplementary file 1 [file jfb-09-00054-s001.zip › SuppMaterial/Fig S2.tif]

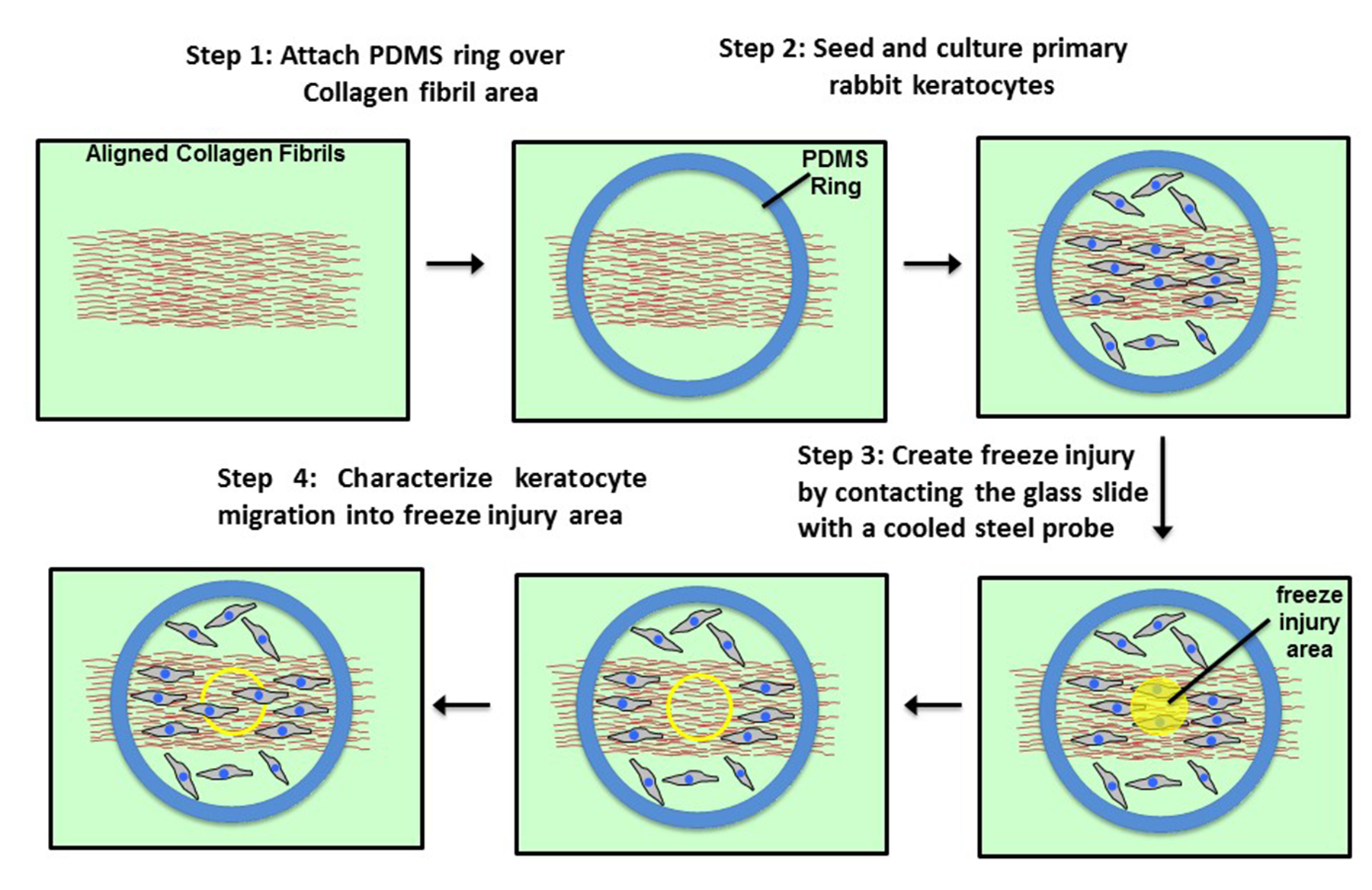

Supplement: Supplementary file 1 [file jfb-09-00054-s001.zip › SuppMaterial/Fig S3.tif]
